# Change of contraceptive preference after the free-LARC program for Thai teenagers

**DOI:** 10.1186/s12905-022-01797-9

**Published:** 2022-06-07

**Authors:** Sathaphone Inthavong, Tawiwan Pantasri, Nuntana Morakote, Tanarat Muangmool, Wirawit Piyamongkol, Saipin Pongsatha, Somsak Chaovisitseree

**Affiliations:** Department of Obstetrics and Gynecology, Faculty of Medicine, Chiang Mai University, Chiang Mai, Thailand

**Keywords:** Contraception, Long-acting reversible contraceptives, Contraceptive implant, Adolescent

## Abstract

**Background:**

In 2014, the Thai government launched a free-of-charge long-acting reversible contraception (LARC) program for Thai female adolescents. However, its acceptance had not been reported. Therefore, this study aimed to describe contraceptive use among women of reproductive age before and after the program was implemented.

**Methods:**

This retrospective cross-sectional study was carried out from the medical records of 9000 women of reproductive age, who attended the Family Planning Clinic at Maharaj Nakorn Chiang Mai Hospital between 2009 and 2018. The Chi-square test was used to compare the contraceptive methods administered before and after the program was implemented, and binary logistic regression was used to find the factors associated with implant use after completion of the program.

**Results:**

Depot medroxy progesterone acetate (DMPA) injection was the most popular contraceptive method used among 40.9% of the women. The rates of subdermal implant use were increased significantly after the program was implemented (2.3–9.3%, *p* < 0.001). Implant use for adolescents aged less than 20 years increased from 2.6% to 56.4%, while DMPA was the most popular method used among adult women at 36.4%. Factors associated significantly with implant use after implement of the program included age of less than 20 years (aOR = 4.17 (CI: 1.84–9.44); *p* = 0.001) and nulliparity (aOR = 8.55 (CI: 3.77–19.39); *p* < 0.001).

**Conclusion:**

This study showed a significant increase in contraceptive implant use after the free-LARC program for adolescents had been applied.

**Statement of implications:**

Contraceptive implant is the most effective hormonal reversible contraception. Its use rate is low among all age groups, but increased clearly after the free-of-charge program was applied for adolescents.

## Background

Contraception is beneficial in maternal and child health improvement, economic growth and education enhancement by spacing pregnancies, limiting family size, and preventing unintended pregnancy. There are many reversible contraceptive methods including natural family planning, the barrier method, combined oral pills, progestin-only pills, transdermal patch, vaginal ring, injected contraception, implants, and intrauterine devices (IUDs) [1]. Long-acting reversible contraceptives (LARC) include the etonogestrel and levonorgestrel subdermal implant, copper-intrauterine device (Cu-IUD) and levonorgestrel-releasing intrauterine systems [2–4]. LARC is the most effective reversible contraceptive method, due to its lowest number of failures and higher continuation rate [2–6]. It was recommended as the first-line contraceptive method for the prevention of unintended pregnancy in adolescents [2, 3]. Nevertheless, The most popular contraceptive used among Thai post-partum women was injected progestin, and the least, implant [7].

Adolescent pregnancy is a public health issue that can cause social, educational and health problems for female adolescents and their offspring. It is associated with an increased risk of maternal and neonatal morbidities [8, 9]. Therefore, an appropriate contraceptive service is needed for all adolescents [10]. The problem of adolescent pregnancy is of concern in Thailand [11], and so the Thai government launched the adolescent pregnancy prevention program in 2014. This program aimed to increase effective contraception for adolescents by offering no cost LARC for Thai women aged under 20 years. The free-LARC program for adolescents was initiated at the Family Planning Clinic, Maharaj Nakorn Chiang Mai Hospital around the end of 2014. Increased LARC use after implementing the free support service has succeeded as previously reported [12–14]. However, contraceptive preference depends on many factors, such as, myth, attitudes, awareness and misperception [3], which differ among ethnicities [15–17].

The objective of the study was to describe contraceptive use among Thai women of reproductive age before and after implementation of the free-LARC program for adolescents.

## Materials and methods

This was a retrospective cross-sectional study conducted at the Family Planning Clinic, Maharaj Nakorn Chiang Mai Hospital from 2009 to 2018. The contraceptive services provided at the hospital included condoms, oral contraceptive pills, injected progestin (depot-medroxy progesterone acetate; DMPA), subdermal implant (LNG and ENG), copper-intrauterine device (Cu-IUD), levonorgestrel IUD, vaginal ring*,* and female and male sterilization. However, the intrauterine device and vaginal ring had limited availability during the study period. The women in this study had to pay for the cost of contraception themselves, except for those aged less than 20 years, who could select the use of implant or Cu-IUD free of charge with support from the government. All of the Thai women who attended the Family Planning Clinic at Maharaj Nakorn Chiang Mai Hospital for the first time between 2009 and 2018 met the inclusion criterion to participate in this study. If they came for more than one visit during that period, they were counted only once. However, if they switched to a different contraceptive method during that time, they would be counted twice.

The exclusion criterion was incomplete data from the medical records. Data of 9,000 women of reproductive age were included in this study, which was approved by the Ethics Committee of the Faculty of Medicine, Chiang Mai University (OBG-2562-06406). The data were collected from medical records. Demographic data and contraceptive choices of the participants were collected**.** All statistical tests were perform with the statistical package for social science (SPSS, USA version 22.0). The Chi square test was used to compare the percentage of contraceptive use before (2009–2014) during and after (2015–2018) the free-LARC program for adolescents. Multivariable logistic regression with backward selection was employed to find the association between the characteristics of implant users after the program. A *p* value of less than 0.05 was considered statistically significant.

## Results

**Fig. 1:**
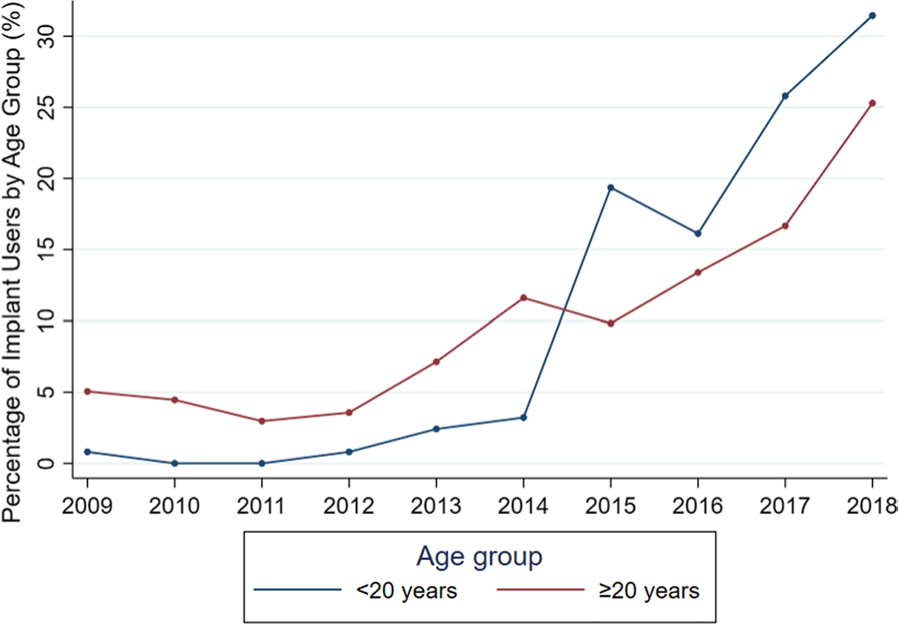
Percentage of women using contraceptive implant between those under and over 20 years of age from 2009 to 2018

Nine thousand women attended to the Family Planning Clinic at Maharaj Nakorn Chiang Mai Hospital from 2009 to 2018. Their mean age was 28.53 ± 6.09 years. Almost all of them (98.5%) were married or had a partner. Eighty-eight percent of these women came to the clinic for postpartum care, while 1,096 of them were non-postpartum. Half of the women (48.4%) were employed in the private sector. Approximately one-third (38.5%) of them had an educational level of bachelor’s degree as shown in Table 1. DMPA injection was the most popular contraceptive method used among 40.9% of the women, followed by progestin-only pills (30.3%). There was an increase of implant use after the free-LARC program for adolescents was applied, while the use of DMPA decreased as shown in Table 2 and Fig. 1.

**Table 1:** Demographic characteristics of women aged younger than 20 years and those aged 20 years and older before and after the free-LARC program for adolescents was applied

Characteristics	Before the program (n = 5388)		*P*	After the program (n = 3612)		*P*	*P*^a^	*P*^b^
	Age < 20 yrs n = 350 (%)	Age ≥ 20 yrs n = 5038 (%)		Age < 20 yrs n = 204 (%)	Age ≥ 20 yrs n = 3408 (%)			
Mean age (y)	17.9 ± 1.3	29.0 ± 6.2		18.2 ± 2.0	29.6 ± 5.5			
**Occupation**			< 0.001			< 0.001	< 0.001	< 0.001
Agriculture	5 (1.4)	92 (1.8)		5 (2.5)	39 (1.1)			
Business	1 (0.3)	169 (3.4)		1 (0.5)	154 (4.5)			
Employee	51 (14.6)	2503 (49.7)		34 (16.7)	1766 (51.8)			
Government officer	1 (0.3)	360 (7.1)		0 (0.0)	312 (9.2)			
Merchant	24 (6.9)	371 (7.4)		7 (3.4)	235 (6.9)			
Housewife/unemployed	179 (51.1)	1411 (28.0)		64 (31.4)	791 (23.2)			
Student	89 (25.4)	132 (2.6)		93 (45.6)	111 (3.3)			
**Education**			< 0.001			< 0.001	0.002	< 0.001
None	26 (7.4)	490 (9.7)		8 (3.9)	275 (8.1)			
Primary school	31 (8.9)	390 (7.7)		18 (8.8)	94 (2.8)			
High school	219 (62.6)	1350 (26.8)		154 (75.5)	786 (23.1)			
Diploma	67 (19.1)	809 (16.1)		22 (10.8)	458 (13.4)			
Bachelor degree	7 (2.0)	1864 (37.0)		2 (1.0)	1593 (46.7)			
Higher degree	0 (0.0)	135 (2.7)		0 (0.0)	202 (5.9)			
**Sexual relationship**			< 0.001			< 0.001	0.029	0.501
Single	9 (2.6)	9 (0.2)		14 (6.9)	10 (0.3)			
Married/cohabiting	331 (94.6)	4989 (99.0)		181 (88.7)	3368 (98.8)			
Divorced/widowed	10 (2.9)	40 (0.8)		9 (4.4)	30 (0.9)			
**Parity**			< 0.001			< 0.001	< 0.001	< 0.001
Nulliparous	12 (3.4)	31 (0.6)		48 (23.5)	90 (2.6)			
Multiparous	338 (96.6)	5007 (99.4)		156 (76.5)	3318 (97.4)			
**Types of visit**			0.417			< 0.001	< 0.001	0.122
Postpartum care	306 (87.4)	4430 (87.9)		122 (59.8)	3046 (89.4)			
Postabortion care	15 (4.3)	267 (5.3)		18 (8.8)	157 (4.6)			
Contraceptive service	29 (8.3)	341 (6.8)		64 (31.4)	205 (6.0)			

^a^*p* Values from the Chi-square test of women aged younger than 20 years before, during and after the program

^b^*p* Values from the Chi-square test of women aged 20 years and older before, during and after the program

**Table 2:** The contraceptive methods used among women aged younger than 20 years and those aged 20 years and older before and after the free-LARC program for adolescents was applied

Characteristics	Before the program (n = 5388)		*P*	After the program (n = 3612)		*P*	*P*^a^	*P*^b^
	Age < 20 yrs n = 350 (%)	Age ≥ 20 yrs n = 5038 (%)		Age < 20 yrs n = 204 (%)	Age ≥ 20 yrs n = 3408 (%)			
**Contraceptive methods**			< 0.001			< 0.001	< 0.001	< 0.001
Combined oral pill	19 (5.4)	295 (5.9)		7 (3.4)	138 (4.0)			
Progestin only pill	80 (22.9)	1508 (29.9)		21 (10.3)	1117 (32.8)			
Condom	12 (3.4)	505 (10.0)		8 (3.9)	450 (13.2)			
DMPA	209 (59.7)	2191 (43.5)		44 (21.6)	1240 (36.4)			
Subdermal Implant	9 (2.6)	117 (2.3)		115 (56.4)	219 (6.4)			
IUD	1 (0.3)	70 (1.4)		0 (0.0)	14 (0.4)			
Vaginal Ring	0 (0.0)	13 (0.3)		0 (0.0)	0 (0.0)			
Male sterilization	0 (0.0)	4 (0.1)		0 (0.0)	3 (0.1)			
Female sterilization	2 (0.6)	54 (1.1)		0 (0.0)	14 (0.4)			
Non-use	18 (5.1)	281 (5.6)		9 (4.4)	213 (6.3)			

^a^*p* Values from the Chi-square test of women aged younger than 20 years before, during and after the program

^b^*p* Values from the Chi-square test of women aged 20 years and older before, during and after the program

After launching the program, implant use among women aged under 20 years clearly increased and became the most popular contraceptive method among adolescents, while DMPA remained the most common contraceptive used among adult women, as shown in Fig. 1 and Table 2. There was a greater proportion of students involved after the program was implmented.

Among 460 women using subdermal implant, the age of less than 20 years and nulliparity were factors associated with implant use after adjustment for other related factors, as shown in Table 3.

**Table 3:** Factors associated with subdermal implant use after the free-LARC program for adolescents was applied (n = 460)

Characteristics	Program		Univariable analysis		Multivariable analysis	
	Before (n = 126) (%)	After (n = 334) (%)	OR (95%CI)	*P*	aOR* (95%CI)	*P*
**Age group**				< 0.001		0.001
≥ 20	117 (92.9)	219 (65.6)	1		1	
< 20	9 (7.1)	115 (34.4)	6.83 (3.34–13.95)		4.17 (1.84–9.44)	
**Occupation**				< 0.001		
Student	12 (9.5)	119 (35.6)	1			
Worker	74 (58.7)	143 (42.8)	0.19 (0.10–0.38)			
Housewife	40 (31.7)	72 (21.6)	0.18 (0.09–0.37)			
**Education**				< 0.001		0.012
None	15 (11.9)	10 (3.0)	1		1	
≤ High school	47 (37.3)	215 (64.4)	6.86 (2.90–16.22)		3.56 (1.39–9.09)	
> High school	64 (50.8)	109 (32.6)	2.55 (1.08–6.02)		2.04 (0.82–5.10)	
**Sexual relationship**				0.110		
Single	2 (1.6)	17 (5.1)	1			
Married/Cohabiting	124 (98.4)	315 (94.3)	0.30 (0.07–1.31)			
Divorced/widowed	0 (0.0)	2 (0.6)	N/A			
**Parity**				< 0.001		< 0.001
Multiparous	118 (93.7)	227 (68.0)	1		1	
Nulliparous	8 (6.3)	107 (32.0)	6.95 (3.28–14.75)		8.55 (3.77–19.39)	
**Types of visit**				0.091		< 0.001
Contraceptive service	70 (55.6)	175 (52.4)	1		1	
Postpartum care	44 (34.9)	143 (42.8)	1.30 (0.84–2.01)		2.45 (1.48–4.07)	
Postabortion care	12 (9.5)	16 (4.8)	0.53 (0.24–1.18)		0.60 (0.23–1.52)	

^*^aOR = adjusted odds ratio with related factors; age group, education, parity, type of visit

The subgroup analysis among the non-postpartum group revealed that more nulliparous and adolescent women attended the clinic after the free-LARC program had been launched, as shown in Table 4. The percentage of implant use among the non-postpartum group rose by nearly 3.5-fold.

**Table 4:** Demographic characteristics and contraceptive methods of postpartum and non-postpartum women before, during and after the program

Characteristics	Before the program (n = 5388)		*P*	After the program (n = 3612)		*P*	*P*^a^	*P*^b^
	Postpartum n = 4736 (%)	Non-postpartum n = 652 (%)		Postpartum n = 3168 (%)	Non-postpartum n = 444 (%)			
Mean age (y) ± SD	27.8 ± 5.5	31.8 ± 8.8		29.0 ± 5.5	28.5 ± 8.8			
**Age Groups**			< 0.001			< 0.001	< 0.001	< 0.001
≤ 19	306 (6.5)	44 (6.7)		122 (3.9)	82 (18.5)			
20–29	2716 (57.3)	227 (34.8)		1614 (50.9)	167 (37.6)			
30–39	1612 (34.0)	264 (40.5)		1346 (42.5)	149 (33.6)			
≥ 40	102 (2.2)	117 (17.9)		86 (2.7)	46 (10.4)			
**Occupation**			< 0.001			< 0.001	< 0.001	< 0.001
Agriculture	54 (1.1)	43 (6.6)		34 (1.1)	10 (2.3)			
Business	143 (3.0)	27 (4.1)		128 (4.0)	27 (6.1)			
Employee	2,283 (48.2)	271 (41.6)		1,616 (51.0)	184 (41.4)			
Government officer	302 (6.4)	59 (9.0)		282 (8.9)	30 (6.8)			
Merchant	328 (6.9)	67 (10.3)		222 (7.0)	20 (4.5)			
Housewife/unemployed	1,441 (30.4)	149 (22.9)		795 (25.1)	60 (13.5)			
Student	185 (3.9)	36 (5.5)		91 (2.9)	113 (25.5)			
**Education**			< 0.001			< 0.001	< 0.001	< 0.001
None	448 (9.5)	68 (10.4)		246 (7.8)	37 (8.3)			
Primary school	322 (6.8)	99 (15.2)		90 (2.8)	22 (5.0)			
High school	1,395 (29.5)	174 (26.7)		768 (24.2)	172 (38.7)			
Diploma	800 (16.9)	76 (11.7)		427 (13.5)	53 (11.9)			
Bachelor degree	1,665 (35.2)	206 (31.6)		1,464 (46.2)	131 (29.5)			
Higher degree	106 (2.2)	29 (4.4)		173 (5.5)	29 (6.5)			
**Sexual relationship**			< 0.001			< 0.001	0.739	0.021
Single	8 (0.2)	10 (1.5)		5 (0.2)	19 (4.3)			
Married/cohabiting	4,684 (98.9)	636 (97.5)		3128 (98.7)	421 (94.8)			
Divorced/widowed	44 (0.9)	6 (0.9)		35 (1.1)	4 (0.9)			
**Parity**			< 0.001			< 0.001	0.017	< 0.001
Nulliparous	4 (0.1)	39 (6.0)		10 (0.3)	128 (28.8)			
Multiparous	4,732 (99.9)	613 (94.0)		3158 (99.7)	316 (71.2)			
**Contraceptive methods**			< 0.001			< 0.001	< 0.001	< 0.001
Combined oral pill	163 (3.4)	151 (23.2)		58 (1.8)	87 (19.6)			
Progestin only pill	1,582 (33.4)	6 (0.9)		1135 (35.8)	3 (0.7)			
Condom	470 (9.9)	47 (7.2)		419 (13.2)	39 (8.8)			
DMPA	2,250 (47.5)	150 (23.0)		1238 (39.1)	46 (10.4)			
Subdermal Implant	44 (0.9)	82 (12.6)		143 (4.5)	191 (43.0)			
IUD	13 (0.3)	58 (8.9)		2 (0.1)	12 (2.7)			
Vaginal Ring	1 (0.0)	12 (1.8)		0 (0.0)	0 (0.0)			
Male sterilization	4 (0.1)	0 (0.0)		3 (0.1)	0 (0.0)			
Female sterilization	1 (0.0)	55 (8.4)		3 (0.1)	11 (2.5)			
Non-use	208 (4.4)	91 (14.0)		167 (5.3)	55 (12.4)			

^a^*p* Values from the Chi-square test of postpartum women before, during and after the program

^b^*p* Values from the Chi-square test of non-postpartum women before during and after the program

## Discussion

Accessibility of social, economic, educational and contraceptive services has impact on the contraception needed and appropriate type chosen [17]. The prevalence of LARC use increased in a decade, but low financial income might be a cause of limited selection [18–20].

The most common contraceptive method used in this study was DMPA. While 87.8% of the women in this study were in the postpartum period, and could not use estrogen during the breastfeeding period, DMPA remained popular among the non-postpartum group. The less popular combination of oral contraceptive pills and condoms might have easier access, as these two products can be bought over-the-counter (OTC) at a pharmacy. The women who attended the Family Planning Clinic at the hospital tended to want less convenient contraceptive methods.

The contraceptive implant is the most effective reversible hormonal contraception method, with a high continuation and satisfaction rate among adolescents and adult women [2–5, 21–23]. The barriers against obtaining this type of contraception includes misperception regarding safety and side effects, fear and pain from the insertion process, and cost of the implant [3]. However, over the last ten years, more women have been interested in using an implant. Despite the dramatic increase in the use of this method among teenagers in this study, the financial issue is an important obstacle for adolescents. Once discarding the financial issue on choice of contraceptive, other studies reported more women selecting the use of LARC, especially, IUD [24, 25]. The obvious increase of implant use has been greater in this study than in previous reports since the free-LARC for adolescents program was implemented [12, 13, 24–26]. This could be explained by the limitation of IUD resources and the postpartum issue. The long-term use of DMPA has had an impact on bone mineral density [3, 27–29], which was not seen in the use of contraceptive implants [30, 31]. More research about bone health and contraception is needed, but in the meantime, the current trend of choice from DMPA to implants for adolescents is a good sign for long-term bone health.

The factors associated with LARC use in general were being married and multiparous, and using to avoid unintended pregnancy and adverse events of short-acting reversible contraception [14, 32, 33]. This study did not explore the reasons for implant use, but after the free-LARC program was implemented, women who were aged less than 20 years and nulliparous had high adjusted odds ratios. Besides the issue of financial support, well-trained health care providers and accessible contraceptive clinics for all women are necessary for appropriate methods of contraception [34–36].

The strength of this study was the large amount of data showing the change of preferred contraception after the free-LARC program had been implemented. The limitations of this retrospective study were limited available data, unavailable uncontrolled resources of IUD and vaginal rings, and OTC access to oral contraceptive pills (OCPs) and condoms. Further study on the awareness and attitude toward LARC is needed for women and health care providers. Extension of the free-LARC program to all age groups might help women select appropriate contraception methods without limitation.

## Conclusion

Implant use increased clearly after the free program was implemented, particularly among women under 20 years of age. It might be assumed that the financial issue is a major barrier for women using contraceptive implants.

## Data Availability

All of the data generated or analyzed during this study are included in the published article.
